# Comparison of Bacterial Microleakage of Endoseal MTA Sealer and Pro-Root MTA in Root Perforation

**DOI:** 10.30476/DENTJODS.2020.86042.1164

**Published:** 2021-06

**Authors:** Mehdi Dastorani, Behnam Shourvarzi, Farshad Nojoumi, Majid Ajami

**Affiliations:** 1 Dept. of Endodontics, School of Dentistry, AJA University of Medical Sciences, Tehran, Iran; 2 School of Dentistry, AJA University of Medical Sciences, Tehran, Iran; 3 Dept. of Microbiology, School of Medicine, AJA University of Medical Sciences, Tehran, Iran

**Keywords:** Dental Leakage, Dental Material, Root Canal Obturation, Endodontics

## Abstract

**Statement of the Problem:**

Different materials have been used to repair root perforations, the most successful of which is mineral trioxide aggregate (MTA). It is technically difficult to use MTA for perforation repair. Recently, some bio-ceramic sealers such as Endoseal MTA were introduced to repair the perforation site during root filling, which decreases the technical difficulty of this procedure.

**Purpose:**

The aim of this study was to compare the bacterial microleakage of Pro-Root MTA and Endoseal MTA sealer in root perforation repair.

**Materials and Method:**

This *in vitro* experimental study evaluated 40 extracted canine teeth. After root canal cleaning and shaping, a root perforation was artificially created at 7 mm below the cementoenamel junction. The teeth were then randomly divided into two experimental groups (n=18) of Pro-Root MTA and Endoseal MTA, and two positive and negative control groups (n=2). Perforation sealing and root canal filling were performed in the two experimental groups according to the manufacturers’ instructions. After sterilization of the whole system with gamma ray, microleakage was tested using a double-chamber model. Data regarding the presence/absence of microleakage were reported after 35 days. The data were analyzed by SPSS software using the Chi-square test.

**Results:**

There was no significant difference between the two experimental groups regarding bacterial microleakage (*p*> 0.05).

**Conclusion:**

Under the conditions of this study, it can be concluded that the sealing ability of perforation repair with Endoseal MTA Sealer and Pro-Root MTA was comparable.

## Introduction

Root perforation is a procedural error that may occur during root canal treatment. Although deep caries or resorptive processes may also cause perforations, most root perforations occur iatrogenically. Root perforations resulting in endodontic treatment failure account for approximately 10% of all failed cases [ 1].

Root perforation refers to a communication between the root canal system and the external root surface [ 2]. Root perforation affects the long-term prognosis of the tooth. The prognosis of endodontic treatment of such teeth depends on the size, position, and time of perforation, as well as the sealing ability of the material used for perforation repair [ 3]. The prognosis would be relatively good if the perforation is quickly detected and sealed with a biocompatible material [ 4]. Various materials have been used to repair the perforation site. An ideal restorative material to repair radicular perforations should be non-toxic, nonabsorbable, radiopaque, and bactericidal or bacteriostatic, and should provide a hermetic seal against microleakage [ 5]. Mineral trioxide aggregate (MTA) was introduced to endodontics in 1990 as a new root-end filling material with the ability to seal the communications between the tooth and the external root surface [ 6- 7]. In addition to being used in root end filling, it is also widely used in other cases such as vital pulp therapy, pulp revascularization, and root perforation repair [ 7- 9]. Assessment of physical, chemical, and biological properties of MTA confirmed its efficacy as a suitable material to seal root perforations [ 7, 10- 11]. Despite the optimal sealing ability and other advantages, long setting time and difficult handling are the main drawbacks of MTA for use in root perforation repair [ 9, 12]. Recently, Endoseal MTA (Maruchi; Wonju, Korea) was introduced to the market, which is an injectable calcium silicate-based root canal sealer. Endoseal MTA has favorable biocompatibility/ odontogenicity comparable to AH Plus (Dentsply DeTrey, Germany), which is a widely used resin-based sealer [ 13]. The manufacturer claims that it has high sealing ability; on the other hand, its clinical application is easier than that of conventional MTA for root perforation repair. Considering the limited number of studies on sealing ability of Endoseal MTA, the aim of this study was to evaluate and compare the bacterial microleakage and sealing ability of Endoseal MTA and Pro Root MTA in root perforation repair. 

## Materials and Method

### Preparation of teeth and perforation repair

The study was conducted at the Department of Endodontics, Dental School, AJA University of Medical Sciences and approved by the Ethics Committee of this university (97000846). A total of 40 freshly extracted single-rooted human canine teeth were evaluated in this *in vitro*, experimental study. The teeth were extracted for reasons not relevant to this research (periodontal or restorative reasons). After collection of eligible teeth, soft and hard tissue residues were removed from the external root surface with a hand and ultrasonic scaler, and the teeth were immersed in 2% sodium hypochlorite solution (Chloraxid, Cerkamed, Poland) for surface disinfection for 30 minutes. After rinsing, the samples were stored in chloramine T solution (Merck, Darmstadt, Germany) until the experiment. Radiographs were taken in buccolingual and mesiodistal directions from all teeth. Teeth with extensive restorations, root caries, immature roots, cracks, internal or external root resorption, or calcified root canals were excluded from the study. The tooth crowns were cut perpendicular to the longitudinal axis 5 mm above the cementoenamel junction with a diamond disc (Diamond, Tehran, Iran), and a standard access cavity was prepared. The working length of each canal was determined by ISO #15 K-file (Mani, Japan) 1mm short of the anatomic apex. After determining the working length of the root canals, cleaning and shaping started with hand filing in working length until ISO #25 K-file (Mani, Japan) and then continued with rotary Neoniti files (Neoniti, Neolix, Fran-ce). For enlargement of the coronal portion, C1 NiTi file (size 25/0.12) was used with brushing movement. The apical two-thirds of the canals were prepared with A1 NiTi file (25/0.08) with brushing movement. According to the manufacturer, endodontic micromotor operating at 300 rpm and 1.5 Ncm torque was used. Each file was used for instrumentation of three canals. The root canals were profoundly irrigated with 2% sodium hypochlorite (Chloraxid, Cerkamed, Poland) after using each instrument. Then, they were rinsed with 17% EDTA (Endo-solution, Cerkamed, Poland). 2mL of distilled water was used for the final rinse of each canal. Next, a perforation was artificially created with 1mm width and 2mm height at 7mm below the cementoenamel junction in the mesial surface of the roots. The perforation was created externally perpendicular to the longitudinal axis of the tooth using a fissure bur to standardize the size of perforation in all teeth. Then, the teeth were randomly divided into two experimental (n=18 in each group) and two control (n=2) groups. The experimental and control groups were prepared as follows.

In the group A, the perforation was repaired with ProRoot MTA (Dentsply, Tulsa, OK, USA). A #40 ISO size gutta-percha point was placed in the canal to maintain its patency during the perforation repair. The ProRoot MTA was prepared according to the manufacturer's instructions, applied at the site of perforation, and burnished. Then, the teeth were incubated at 37°C for 24 hours, and the canals were filled with AH-26 sealer and 2% gutta-percha points (Meta, South Korea) using the lateral compaction technique.

In the group B, the perforation was repaired with Endoseal MTA sealer, and the canals were filled according to the manufacturer’s instructions. The teeth were then incubated for 24 hours similar to group A.

In the group C, samples served as the positive control, which received no restorative material at the perforation site or in the root canal. The next steps were carried out as in other groups.

In the group D, samples served as the negative control with no perforation. The entire access cavity was filled with sticky wax (Pyrax, India). The next steps were carried out as in other groups.

**Figure 1:**
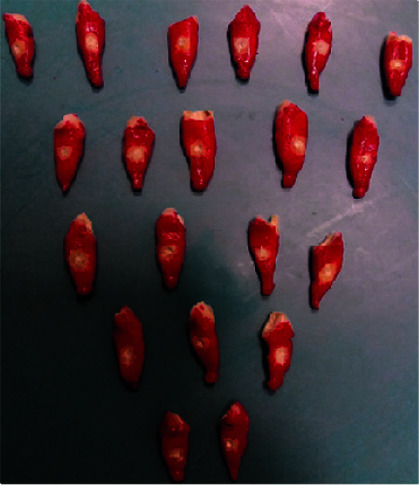
Applying two layers of nail varnish over the specimens to block the pathways of bacterial penetration through dentinal tubules and accessory canals

The groups were coded such that the examiner was blinded to the type of treatment and the material used for perforation repair. The teeth were then sealed with composite resin (2 mm at the root end) to prevent microleakage through the apex. The entire root surface, except for the perforation site and 1mm margin around the perforation site, was coated with two layers of nail varnish (Isadora, Sweden) to prevent bacterial microleakage through the dentinal tubules and accessory canals (Figure 1). In the positive control group, nail varnish and composite application was performed as in groups A and B, except that the access cavity was directly connected to the outer root surface via the perforation. In the negative control group, the entire root surface was covered with two layers of nail varnish and the access cavity was filled with sticky wax.

### Bacterial microleakage test

A double-chamber system was designed and used to evaluate bacterial infiltration.

**Figure 2:**
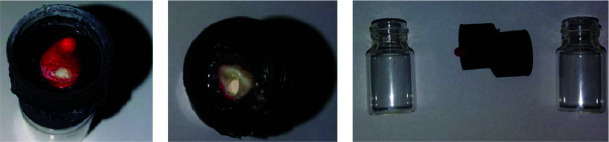
Double-chamber system: The tooth was fixed at the center of the cap using cyanoacrylate and aquarium glue to prevent bacterial penetration from the top

This system contains an upper chamber for bacterial Inoculation and a lower chamber for the sterile substrate, and the tooth was interposed between them such that the perforation site was located in the lower chamber (Figure 2). All the communication paths between the upper and lower chambers were sealed with cyanoacrylate and aquarium glue (Caspian, Iran). Next, the double-chamber system was sterilized by gamma radiation (13 hours, 25 kGy). Azide dextrose broth culture medium was injected into the lower chamber such that the perforation site was positioned in the solution and sterilized again by gamma radiation.

*Enterococcus faecalis* (E. *faecalis*) were obtained from Pasteur Institute, Tehran, Iran. In order to reach accurateness of bacterial purity, they were first cultured in explicit media. Pure bacteria colonies were derived from blood agar and cultured in 10cc TSB and were then incubated in 37˚C for 24 hours so that a 0.5 McFarland (1.5×10^8^ bacteria/mL) solution concentration were yielded. Using a sterile syringe, 0.1mL bacteria was inoculated into the pulp chamber of the teeth every 48 hours and then the samples were incubated at 37°C. Specimens were examined over a period of 35 days, during which the teeth were kept in an incubator and checked daily. The turbidity of the culture medium indicated bacterial microleakage. At the end of the experiment, the bile esculin combined with 6.5% NaCl tolerance test was performed to confirm the bacterial infiltration in case of culture medium turbidity [ 11- 15]. Assessments were made by a person blinded to the type of sealing material used. Data were analyzed using SPSS version 19 via the Chi-square test. All statistical tests were performed at the significance level of the *p*< 0.05.

## Results

**Figure 3:**
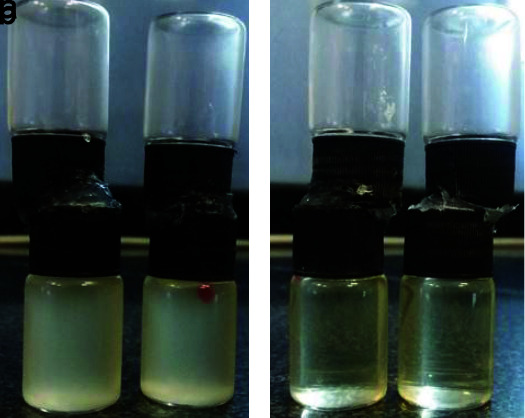
**a:**In all teeth in the positive control group, **b:** Bacterial microleakage was observed 24 hours after the onset of the experiment. However, it was not observed in any of the negative control samples.

Table 1 present the bacterial microleakage data in the experimental groups. In Endoseal MTA group, no bacterial microleakage was noted after 35 days. In Pro-Root MTA group, three (17%) samples showed bacterial microleakage; two of which occurred at 10 days and one occurred at 12 days after the onset of the experiment. However, the Chi-square test revealed no significant difference between the two experimental groups (*p*> 0.05). Bacterial microleakage was observed in all positive control teeth 24 hours after the onset of the experiment. However, bacterial microleakage did not occur in any of the negative control teeth (Figure 3). 

**Table1:** Comparison of bacterial microleakage percentage between Endoseal MTA and Pro-Root MTA groups

		Endoseal MTA	Pro-Root MTA	*p* Value
Microleakage	Yes	0(0%)	3(17%)	0.07[Table-fn t1f1]*
	No	18(100%)	15(83%)	

"*" Indicates a significant difference with *p*<0.05. Data were expressed as frequency (percentage) and analyzed by the Chi-square test.

**Figure 4:**
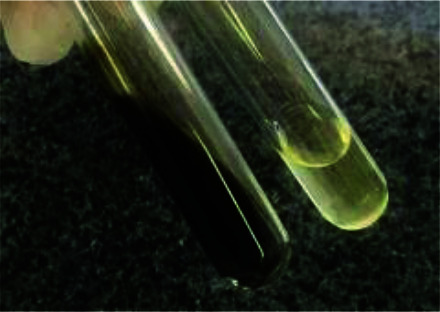
Bile esculin combined with 6.5% NaCl tolerance test to confirm the presence of bacterial colonies in cases with turbidity

The results of bile esculin combined with 6.5% NaCl tolerance test as well as Gram staining confirmed the microleakage of *E. faecalis* into the culture media that showed turbidity (Figure 4). 

## Discussion

Different materials have been used to repair root perforrations [ 14]. MTA is the most commonly used material for root perforation repair [ 15]. However, it is technically difficult to use MTA for perforation repair.

Endoseal MTA supplied in airtight syringes and injected into the root canal system. It is self-cure and gradually sets when exposed to air by absorbing the ambient moisture without requiring any mixing. According to the manufacturer, this calcium-silicate cement can be considered as a MTA-derived material because its chemical composition resembles that of MTA. Therefore, it is expected to have favorable physical and biological properties similar to other MTA-derived materials reported in previous studies [ 16- 18]. Furthermore, this injection-type, self-setting root canal sealer has a user-friendly application, which is a clinical advantage [ 13]. According to the manufacturer, this sealer can be used to seal root perforations. Therefore, in this study, we compared the perforation sealing ability of this sealer with Pro-Root MTA, which is the most acceptable material for perforation seal. Bacterial microleakage is the cause of most endodontic infections, so in this study microleakage test was performed using the bacterial infiltration technique. In this study, we used *E. faecalis* because it seems to play a significant role in the etiology of persistent periradicular lesions [ 19]. It should be noted that bacterial microleakage test, although is more similar to clinical conditions than other methods such as dye penetration test [ 20], it has limitations when evaluating the sealing ability of materials which have antibacterial properties such as Pro-Root MTA and Endoseal MTA that were used in this study.

In this study, we evaluated microleakage over a period of 35 days; in other studies, periods of 30 days or more were used [ 6, 21- 22]. The results of our study showed that none of the teeth sealed with Endoseal MTA had bacterial microleakage. However, 17% of the teeth sealed with Pro-Root MTA showed bacterial microleakage. The Chi-square test revealed no significant difference between the two groups in terms of bacterial microleakage.

Khatib *et al*. [ 23] compared sealing ability of Endocem MTA and Endoseal MTA in furcal perforation repair using dye penetration test and showed that Endoseal MTA had significantly lesser sealing ability compared to Endocem MTA. In our study, the Endoseal MTA showed a high sealing ability, the difference between our study and their study can be due to differences in the type of leakage test and the location of the perforation. The washout and dislodgement resistance of repair materials in the furcal and root perforation is different and this issue is more important in the furcal area due to its proximity to the pressures and flow of liquids. This explanation is supported by the study of Adl *et al*. [ 24] that indicated EndoSeal MTA had significantly lesser bond strength than ProRoot MTA and Biodentine. 

De Dues *et al*. [ 25] evaluated 36 furcal perforations during a period of 50 days and found no statistically significant difference between the teeth repaired with Portland cement and those repaired with MTA using bacterial liquid filtration technique. In their study, 50% of the MTA samples and 60% of Portland cement samples had leaked. The MTA used in this study was Pro-Root MTA. Despite the difference in the location of perforation in our study and their study, the number of samples was similar; however, they reported higher percentage of bacterial microleakage than our study. The difference in duration of the two studies can probably explain the difference in the results. Application of Pro-Root MTA is technique-sensitive, and success of treatment highly depends on the skills and experience of the operator. This may justify the difference between their results and ours.

Hwang *et al*. [ 26], compared the bacterial microleakage of Endoseal MTA, Gutta-Flow and AH Plus in root canal obturation of 60 premolar teeth using a laser scanning microscope and reported that Endoseal MTA had significantly higher sealing ability than the other two groups. Although they used a different method for evaluation of bacterial microleakage, their results confirmed the superiority of Endoseal MTA. The absence of microleakage in the group sealed with Endoseal MTA is in line with the results of our study. However, the teeth in our study were different from theirs.

We anticipated less bacterial microleakage in Pro-Root MTA group compared to the Endoseal MTA; but the results proved otherwise. Bacterial microleakage was observed in all teeth of the positive control group and none of the teeth in the negative control group, which confirms our correct methodology. On the other hand, the time interval between the occurrence of bacterial microleakage in the positive control and Pro-Root MTA groups (two samples) indicated the significant effect of Pro-Root. Compared to Endoseal MTA, the application of Pro-Root MTA is much more complicated since it requires more steps. Therefore, the operator’s experience and skills can greatly influence the success of treatment with Pro-Root MTA. This may explain the higher frequency of bacterial microleakage in Pro-Root MTA group.

If the study period was longer, different results could have been obtained. Therefore, future studies over longer periods are required to obtain results that are more reliable. Our results only indicated the presence or absence of bacterial microleakage and did not quantify it. Future studies are recommended to assess the microleakage rate at different time intervals to obtain results that are more accurate. Our study focused solely on the canine teeth; other types of teeth should be evaluated in future studies. This study focused solely on the occurrence of perforation. Therefore, the perforation size was standardized in all teeth. The efficacy of Pro-Root MTA and Endoseal MTA for sealing of different sizes of perforations should also be investigated. 

## Conclusion

Within the limitations of this study, the results showed no significant difference in bacterial microleakage between Pro-Root MTA and Endoseal MTA for root perforation repair. However, Endoseal MTA has easier application and lower technical sensitivity for perforation sealing, which can affect the treatment success. Further studies are required to better elucidate this topic.
